# miR-20a-5p mediates hypoxia-induced autophagy by targeting ATG16L1 in acute kidney injury

**DOI:** 10.1186/cc14018

**Published:** 2014-12-03

**Authors:** I-K Wang, C-Y Li

**Affiliations:** 1 Graduate Institute of Clinical Medical Science, China Medical University, Taichung, Taiwan; 2 Division of Nephrology, China Medical University Hospital, Taichung, Taiwan; 3 Department of Anesthesiology, China Medical University Hospital, Taichung, Taiwan

## Introduction

Autophagy could be induced under stress conditions, including starvation, infection, and hypoxia. The microRNA (miRNA) network may be critical in the regulation of autophagy. Upregulation of autophagy may be a protective response for cell survival in ischemic kidney injury. The aim of this study was to evaluate whether miRNA regulates autophagy in ischemic kidney injury and renal proximal tubular cells under hypoxic conditions.

## Methods

Ischemic kidney injury was performed by clamping bilateral renal pedicles for 60 minutes in male mice. Human kidney proximal tubular (HK2) cells are incubated in a hypoxic chamber with 0.3% O_2_. Bioinformatics analyses were used to select the candidate miRNA. Gain-of-function and loss-of-function methods were employed to evaluate the effects of miRNA on autophagy. Chromatin immunoprecipitation analyses and promoter luciferase reporter assays were used to evaluate the interaction of transcriptional factors with miRNA.

## Results

Increase of LC3 and ATG16L1, autophagy-related proteins, and down expression of miR-20a-5p were detected in kidneys after ischemic injury and in HK2 cells under hypoxic conditions. The 3'-untranslated region luciferase reporter assays indicated that miR-20a-5p targeted ATG16L1 messenger RNA. Overexpression of miR-20a-5p reduced the expression of LC3-II and ATG16L1 in HK2 cells under hypoxic conditions, whereas antagomiR-20a reversed the inhibition. Using RNAi against hypoxia-inducible factor-1α (HIF-1α) in HK2 cells, we confirmed the inhibitory binding of HIF-1α to miR-20a-5p.

## Conclusion

The signaling axis of HIF-1α, miR-20a-5p, and ATG16L1 in autophagic process might be a critical adapting mechanism for ischemic kidney injury.

